# Use of bioaerosol stakeholder mapping and engagement for the development of future strategic collaborations: a UK perspective

**DOI:** 10.1099/mic.0.001574

**Published:** 2025-07-16

**Authors:** Sophie A. Mills, Sana G. Shetty, Gillian H. Drew, Kerry A. Broom, Emma Marczylo, Philippa Douglas

**Affiliations:** 1 Cranfield Environment Centre, Environmental Sustainability Theme, Faculty of Engineering and Applied Sciences, Cranfield University, College Road, Wharley End, Bedford, MK43 0AL, UK; 2 School of Geography, Earth and Environmental Sciences, University of Birmingham, Birmingham, B15 2TT, UK; 3 Radiation, Chemical, Climate and Environmental Hazards, UK Health Security Agency, Chilton, Didcot, Oxfordshire, OX11 0RQ, UK; 4 NIHR Health Protection Research Unit in Chemical Radiation Threats and Hazards, London, UK; 5 NIHR Health Protection Research Unit in Environmental Exposures and Health, London, UK; 6 NIHR Health Protection Research Unit in Environmental Change and Health, London, UK; 7 Centre for Environmental Health and Sustainability, University of Leicester, Leicester, UK; 8 Chief Scientist’s Group, Environment Agency, Red Kite House, Wallingford, UK

**Keywords:** bioaerosols, stakeholder analysis, stakeholder engagement, stakeholders, strategic collaborations

## Abstract

Stakeholder mapping is a process that involves identifying, characterizing and visualizing the connections between stakeholders. It is important to understand the main influencers and those with an interest in or affected by a certain project or field. Stakeholders can have diverse perspectives and priorities, some complementary and others conflicting, and it is important to develop effective engagement strategies to facilitate progress with the desired impact. Here, we present the first such analysis for the bioaerosol research field. This study presents data from a survey and outcomes from workshops attended by researchers with an interest in bioaerosols. Bioaerosols are airborne particles from biological origin that can have diverse and serious effects on public health. Stakeholders were identified and analysed in terms of sector, engagement type, interest and influence scores, expertise and the nature of the relationships between them. Limitations of this study are discussed with suggestions for improvements. The workshops included discussions on missing stakeholders, skills and knowledge gaps, the uses of stakeholder mapping and how to facilitate skill sharing, collaboration and effective progress in the future. We share this to provide a simple but successful approach for researchers with no previous practical experience in stakeholder mapping to modify, use and realize similar benefits within their own fields.

## Data Availability

The data supporting the findings of this study are not publicly available due to privacy and ethical restrictions. No new tools, software or code have been generated.

## Introduction

Bioaerosols are ubiquitous in the atmosphere globally in both outdoor and indoor environments. They are particles suspended in the air originating from living organisms and include pollen, fungi, bacteria, viruses, house dust mites, pet dander, plant debris, algae, fern spores and their constituents and products. Bioaerosols comprise a natural component of airborne particles that are essential for biological processes on Earth, including species colonization, biodiversity and gene dispersal. A recent UK government blog highlights the importance of biological diversity in ambient air, and the benefits of this to health, particularly in developing and maintaining a healthy immune system [1]. However, they can also have serious implications on public health, with exposure to high levels of fewer pathogenic organisms leading to infection and/or allergic conditions such as hay fever, allergic rhinitis and asthma [2,3]. In the UK, allergic rhinitis affects 10–15% of children and 26% of adults, and asthma affects ~9% of children and 17% of adults [4,7]. Some bioaerosol components are known pathogens causing many serious illnesses; for example, *Aspergillus fumigatus* can cause allergic bronchopulmonary aspergillosis in individuals who will alter lung function, such as those with asthma or chronic obstructive pulmonary disease [8,9]. It has recently been estimated that there are 6.5 million invasive fungal infections globally every year and 11.5 million sufferers of fungal asthma [9], with ~20 and 6% of the worldwide population sensitized to pollen or fungi [10,11]. Fungi are also known to produce mycotoxins which, on repeated inhalation, can cause pneumonitis, chronic fatigue, kidney failure and cancer [12]. Recently, the importance of indoor exposure to bioaerosols has become more prominent due to the tragic death of Awaab Ishaak, a 2-year-old boy who died of a severe respiratory condition due to indoor mould exposure [13]. Exposure to damp and mould indoors has been associated with ~5,000 new cases of asthma (~2,200 disability-adjusted life years lost) [14,16]. However, there are no established dose-response relationships for bioaerosols, which can be attributed to several factors including (i) a lack of characterization of different components in ambient air and standardized monitoring methods suitable to assessing exposure over longer time periods to allow comparison of different studies [17,20], (ii) an improved understanding of human exposures in different environments [19,21, 22] and (iii) a lack of toxicological studies that are essential to better understanding the sensitization process and symptom severity [23]. These were also highlighted in recent multidisciplinary workshops that brought together experts to identify and prioritize key gaps in bioaerosol exposure assessment and associated cellular and molecular mechanisms driving health outcomes [24]. Work in this area is vital now more than ever, due to a changing climate. Rising temperatures as a result of climate change are associated with earlier seasons and a higher abundance of some pollen and fungal spores, which may increase the future disease burden of allergy [25].

Given the multidisciplinary nature of bioaerosols, a holistic, system-thinking approach is essential for generating high-quality and reliable evidence to enable governments to develop effective policies and successfully implement regulations that ensure a healthy environment and to improve public health literacy. This requires knowledge exchange and collaboration between government, academia, industry, healthcare practitioners, patients and other stakeholders, including groups unaware that they are part of the system (for example, the general public, as not all will be sufficiently informed on how their daily lives may be affected by the biological composition of the air they breathe). Stakeholders come with diverse perspectives and priorities that can be complementary or conflicting, and progress can be hindered by a limited understanding of the full picture. It is therefore important to invest time in identifying all relevant stakeholders, mapping their interactions and relationships and facilitating active engagement to ensure that all relevant information and opportunities are well communicated and accessible and mitigate potential conflict [26]. The value of investing time in more comprehensive stakeholder mapping is being increasingly recognized across scientific research. For example, chemical engineers interested in adaptation to climate change [27], researchers in ecosystem services [28] and clinicians and public health specialists exploring how community engagement and involvement benefitted the COVID-19 pandemic response [29] have used a variety of methods for identifying and analysing the importance and diverse perspectives of their stakeholders. However, few microbiological and aerosol researchers have practical experience of stakeholder mapping. Leal Filho *et al*. [30] have demonstrated how collaborative efforts between higher education institutions and public communities can contribute to sustainable development and achievement of the UN Sustainable Development Goals. Similar opportunities are possible within the bioaerosol field, whereby the facilitation of improved interactions between different disciplines and sectors will drive successful and impactful partnerships and collaborations for the benefit of wider society.

Very little work to date has been conducted on bioaerosol-specific stakeholder engagement and mapping. In this study, we have presented the first such analysis for the bioaerosol research field. We used data derived from surveys and workshops to (i) identify the key stakeholders and the sector that they represent, (ii) explore reason(s) for engagement, (iii) assess the level of stakeholder importance and interest to help prioritize stakeholders, (iv) survey existing expertise and desired needs of stakeholders to encourage cross-disciplinary collaboration and identify skills gaps and (v) map existing connections and relationships between stakeholders. Through this exercise, we aimed to evaluate feedback from existing stakeholders on both the map and its use, uncovering any missing stakeholders and considering strategic collaborations to facilitate future progress.

## Methods

### Questionnaire

The data for this study are primarily collected from an electronic questionnaire created using Microsoft Forms (Material SA, available in the online Supplementary Material) that was distributed via BioAirNet, a Clean Air Programme-funded network [31], other UK clean air networks [32] and contacts of the authors. The authors also distributed the questionnaire at meetings and conferences and encouraged others to share within their own networks to capture a varied range of stakeholders.

Individuals who responded to the questionnaire are now referred to as respondents, and the organizations or individuals who were identified in the questionnaire are referred to as stakeholders. Some respondents were also identified as stakeholders. Most respondents (51%) were from academic institutions, 19% from industry/private companies, 16% from UK government institutions, 7% from international non-profit organizations (NPOs) and 5% from non-UK government institutions. One respondent identified as a member of the public; however, ‘the public’ was not included as a sector in the following analysis, as insufficient representation was identified. The wider distribution of stakeholders identified by these respondents is explored in the ‘Results’ section.

Respondents to the questionnaire were asked to input all bioaerosol-related stakeholders that they were aware of (whether they had existing contacts/collaborations or not). For each stakeholder, respondents were asked to provide information about the stakeholder, e.g. name of organization, the sector that the organization represented, current and future engagement types (Table 1) and reason for and frequency of engagement, and to provide an opinion on the influence and interest as stakeholders for bioaerosols (scored 1–4, low to high) (Material SA). All respondents who completed the questionnaires contributed voluntarily and were aware that their input would be used for the purpose of this study.

**Table 1.** Definitions of the stakeholder engagement types using the 9Cs model (adapted from NHS England and NHS Improvement [46])

Engagement type	Definition
**Commissioner**	Those who pay for projects to happen.
**Customer**	Those who acquire and use the products or outcomes.
**Collaborators**	Those with whom the organization works to develop products.
**Contributors**	Those from whom the organization acquires content for products.
**Channels**	Those who provide the organization with a route to a market or customer.
**Commentators**	Those whose opinions of the organization are heard by customers and others.
**Consumers**	Those who are served by our customers: i.e. patients, families and users.
**Champion**	Those who believe in and will actively promote the project.
**Competitors**	Those working in the same area who offer similar or alternative services.

### Data cleaning and analysis

The data underwent a thorough cleaning process to prepare it for analysis, which ensured that all entries were consistent, sourced any missing information and established the stakeholders identified as either representing organizations as a whole or specific individuals within an organization. As well as identifying organizations, respondents also identified individual stakeholders within these organizations in the questionnaire, but it was ensured that each organization was represented only once. All stakeholder organizations identified were defined within one of the following categories: academic, national government, local government, industry, NPOs (including charities), societies and networks.

Quantitative analysis and visualization were performed using Python 3.9.7 with standard packages: NumPy (1.23.5), pandas (1.5.3), Matplotlib (3.6.3), Seaborn (0.12.2) and Plotly (5.11.0).

### Workshops and follow-up questionnaire

Respondents who completed the questionnaire were invited to participate in two 2 h follow-up workshops (on the ninth and sixteenth of October 2023), as an opportunity to get feedback and more detailed information, particularly on their perspectives and expertise. The workshop invitation and agendas are provided in Material SB. The workshops were held virtually using Microsoft Teams. All participants of the workshops contributed voluntarily and were aware that their input would be used for the purpose of this study.

At the first workshop, we provided an introduction, outlined the purpose of the workshop and presented the initial, anonymized, results of the questionnaire. After a short break, we facilitated discussions in four breakout rooms. There were seven to eight participants per breakout room, and the attendees assigned to each breakout room were determined in advance to ensure a similar distribution of sector representatives across the breakout rooms. Breakout room discussions were focussed on the following questions:

1. What organizations were missing from the stakeholder map?
2. What further information would be useful to collect from existing stakeholders?
3. How the stakeholder map could be useful?

Google Jamboards were also used for workshop attendees to anonymously record their comments. A follow-up questionnaire was also introduced for them to complete prior to the second workshop.

The follow-up questionnaire (Material SC) was distributed to the workshop invitees. The questionnaire was created using Qualtrics and collected information on stakeholder interests concerning bioaerosols (e.g. measurement and characterization and risk assessment), which bioaerosol types/components they were specifically interested in, and the perceived influence and interest towards bioaerosols from (a) the perspective of the organization that they represented and (b) their perspective as professional individuals. Stakeholders were also asked to define their own areas of expertise, the expertise available to them within their organizations and the expertise that they lacked but would be useful and desirable to them for their work. We also asked three open-ended questions:

1. What are the primary knowledge gaps concerning bioaerosols and important aspects to focus on to facilitate future progress?
2. What questions are important to have amongst stakeholders?
3. What would you like to gain from stakeholder mapping and engagement?

At the second 2 h workshop, in addition to reflections from the previous workshop, the findings of this second questionnaire were presented to the stakeholders to share knowledge and gather feedback. Following a short break, the responses from the second survey led to two more questions being posed to delegates for discussion in two breakout rooms:

1. How do we facilitate skills sharing and collaboration and create a roadmap for the future?
2. How do we engage with the public, health practitioners and any other missing stakeholders to increase awareness and interest?

Once again, Google Jamboards were used so that workshop attendees could anonymously record their comments.

### Mapping stakeholder relationships

To help visualize the connections between stakeholders identified from the questionnaires and workshops, two approaches were used. Kumu [33] was used to create a shareable network map using the data from the questionnaires and workshops. Individual stakeholders were listed as elements, and the relationships between them were listed as connections. The stakeholder sectors were colour-coded, and layers were added to the map to allow the user to view specific sectors only if required. Relationships between stakeholders were categorized using the 9Cs approach (Table 1) and also colour-coded.

As the stakeholder map created in Kumu is visually ‘flat’, this was complemented by an onion diagram, which illustrated the layering of stakeholders in the context of bioaerosols based on their characteristics and connections. The onion diagram made the hierarchical structure and connections amongst stakeholders easier to understand [34] and was used to better comprehend dependencies, connections and hierarchies. Three concentric layers were used to represent different stakeholder groups as follows:

- Inner circle: key players. These were stakeholders with a high level of interest and influence. It is suggested that engaging aggressively with this group should be prioritized in order to impact change [35].
- Mid-layer: subjects. These were stakeholders with high interest and wanting to influence but having little power. They must be kept interested and involved [36].
- Outer layer: crowd. These were stakeholders with the lowest influence and interest. This group of stakeholders should be informed about significant events and outcomes [37].

## Results and discussion

### Original questionnaire

#### Geographical and sector representation

**Fig. 1.**
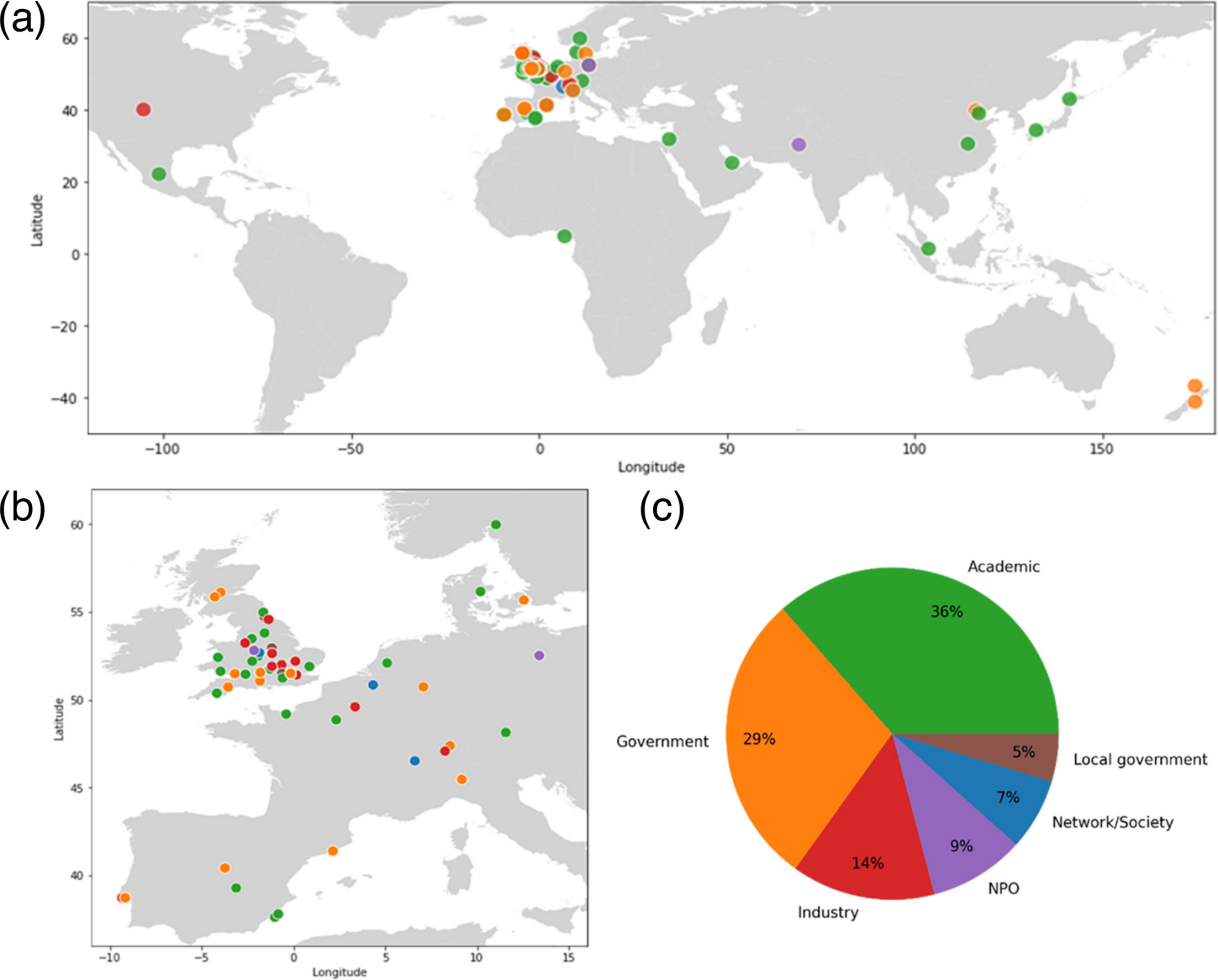
(a) A map depicting where stakeholders were based globally, (**b**) within the UK and Europe and (c) the proportion of stakeholder organizations within each sector. The colours in (a) and (b) correspond with the sector colours in (c).

Our initial survey was completed by 55 people from 39 organizations. They identified a total of 129 different stakeholder organizations and 93 individuals from amongst these organizations. The majority (64%) of organizations identified were based in the UK, 25% from other European countries and 11% from non-European countries (Fig. 1a, b). The majority of stakeholder organizations were based in academia (36%), 29% in government, 14% in industry and 21% in other sectors (Fig. 1c). A similar pattern was seen for individual stakeholders, whereby the majority were from academia (62%), a quarter (25%) from government and only 12% from other sectors. This is likely reflective of our own wider networks, being UK-based research scientists within academia and government ourselves.

#### Engagement types

**Fig. 2.**
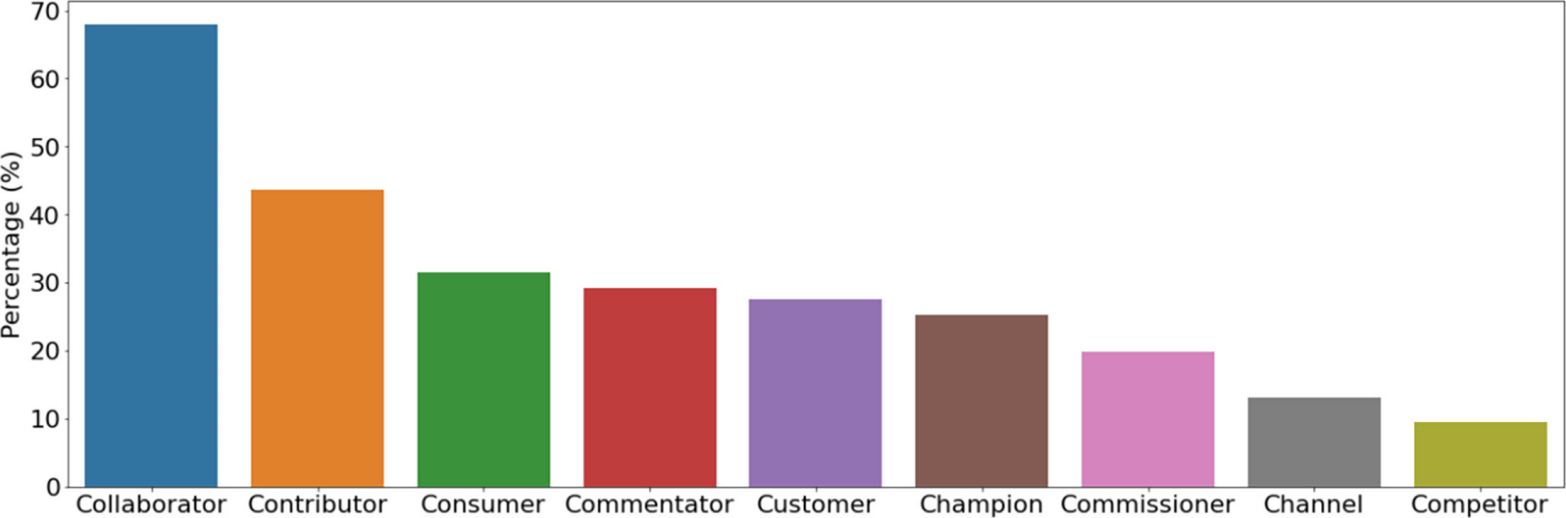
Percentage of stakeholders (organizations and individuals) who identified with each engagement type. The stakeholder engagement types are defined in Table 1.

Fig. 2 shows the percentage of stakeholders (organizations and individuals combined) who identified with each engagement type (Table 1). Nearly 70% were described as ‘collaborators’, i.e. those who are directly involved in projects for developing knowledge, services and products, and largely comprised of researchers in academia and government. This highlights that there are already significant positive relationships between existing stakeholders within the bioaerosol field, probably the result of the bioaerosols being an area predominantly comprised of a relatively small number of researchers who regularly collaborate on bioaerosol research. Between 25 and 50% of stakeholders were contributors, consumers, commentators, customers and champions – i.e. those that acquire resources, services or products; directly or indirectly use services or products; or actively promote the field/project. Customers and consumers in this case included government agencies, companies, charities and networks that are interested in research projects and developing outputs for their own interest or to benefit the consumers that they serve. Champions included some high-profile, highly engaged individuals from particular academic and government institutions, but also charities and companies invested in the health of their customers and consumers, and networks and societies across Europe interested in environmental and public health. Commentators also included some high-profile individuals from academic institutions, some government departments and learnt societies and networks. This suggests that whilst existing bioaerosol research collaborations and networks are already making some impact, there is scope for further expansion of these connections and mobilization of knowledge.

The less represented types were commissioners, channels and competitors. Commissioners generally included local governments, governmental organizations (such as those invested in business and infrastructure), charities and research councils, whilst channels mainly included governmental organizations, networks and charities. These stakeholders are potential targets for future engagement, with potential interest and resources for specific projects. The few competitors came from NPOs, networks and industries, particularly those that have other priorities such as planning and construction of dwellings that may conflict with the consideration of bioaerosol health concerns. Some academic and governmental organizations from countries outside the UK were also considered as competitors here due to potential conflicting/competing priorities, interests and funding sources. Improved future communication and engagement between these stakeholders, facilitated by the activities described here, may help increase appreciation of different perspectives within a shared common goal and facilitate collaboration rather than competition to secure joint funding that enables all to work towards this goal whilst meeting their own priorities. It would also encourage researchers to cross sectors as well as disciplines to find others to work with beyond their existing network, for example, developing interventions such as improved ventilation and smart buildings with industry partners to make real-world changes. The higher percentage of UK-based stakeholders here also emphasizes the need to develop international collaborations to tackle the challenges associated with bioaerosols under changing climates. This could change these international stakeholders from competitors to collaborators.

#### Interest and influence

**Fig. 3.**
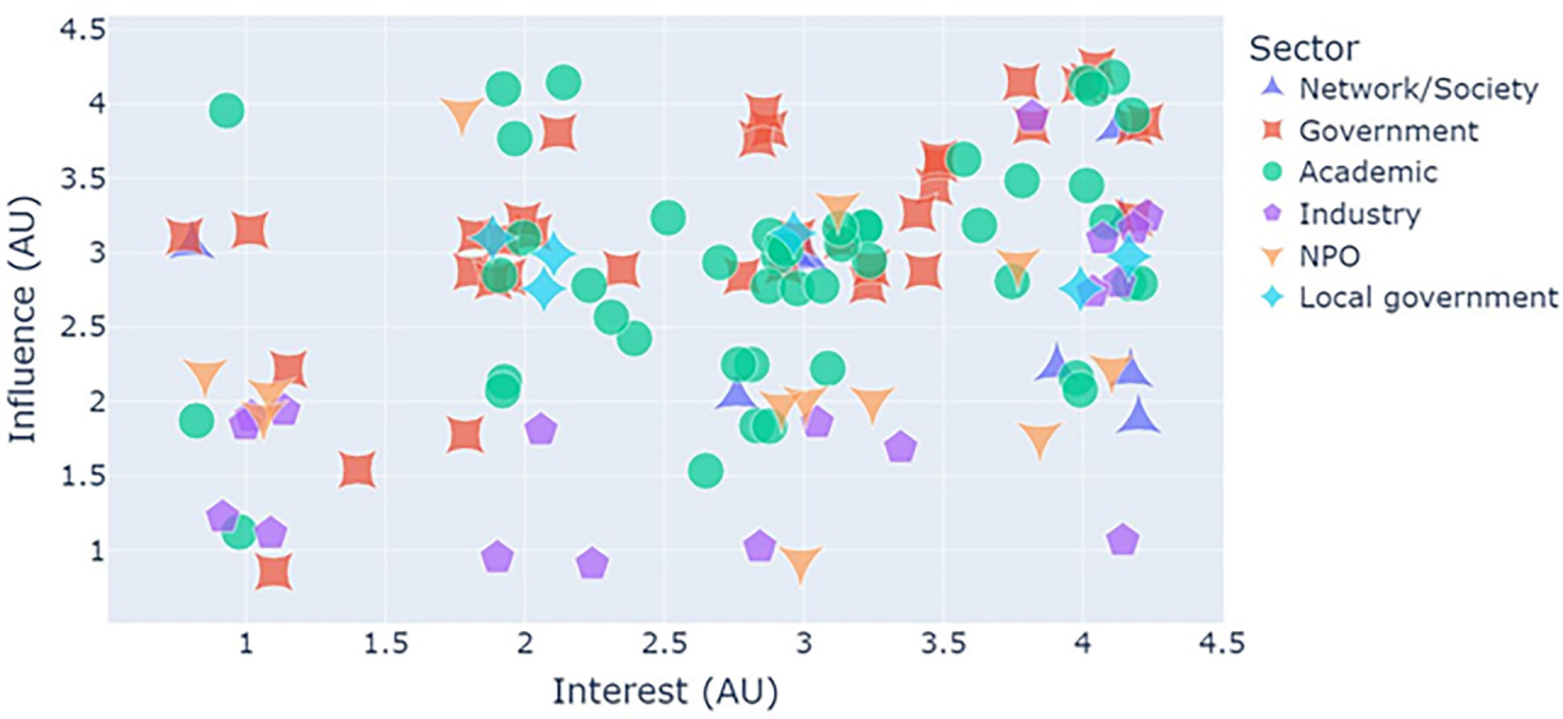
Perceived influence vs interest for stakeholder organizations captured in the original questionnaire. Stakeholders are coded by their sector. Data points have been jittered (to random extents by ±0.25) to aid visualization.

Fig. 3 presents the perceived interest and influence scores of stakeholders, on a scale of 1 (low) to 4 (high), as reported in the original questionnaire stakeholders completed (Material SA). The largest proportion of stakeholders (49%) resided in the top-right quadrant of both high interest and high influence. This included stakeholders across all sectors, but the majority were from government organizations with a remit of environmental and public health and academic institutions with active research programmes in aerosol science, aerobiology, microbiology and environmental public health. There were relatively few NPOs and charities here. This also likely reflects our wider immediate networks as government and academic researchers actively involved in bioaerosol research and highlights the value and impact of collaborations between academic and government research organizations.

Those with low influence but high interest (17%) included academic institutions and NPOs/charities, networks/societies and industries. These stakeholders generally have an interest in aerobiology, allergy, health (particularly respiratory health), and the environment but were perceived to lack influence in the field.

The low-influence, low-interest quadrant (16%) is dominated by industries and NPOs with interests in infrastructure, planning and construction. There were also some academic and government institutions who perhaps have other priorities and consider bioaerosols as a peripheral interest.

Perhaps the most important quadrant, in terms of future engagement, is the high-influence, low-interest group, which was comprised of 17% of stakeholder organizations. This quadrant was dominated by government and academic institutions, many of which were based outside of Europe and particularly in Asia. Within the UK, such stakeholders also included governmental institutions with more general interest in public health and infrastructure (that may be less aware of the impact of bioaerosols but would be very influential in implementing change if they better understood our field). Of note, local governments predominantly resided in this quadrant, as did funding bodies with broad remits. It is clear to see that developing future engagement strategies, including improved knowledge exchange and mobilization, with these stakeholders would help drive new pathways to impact such as developing and implementing policy-based interventions to benefit public health.

### Workshops and follow-up questionnaire

Respondents, representing academia, government institutions and industry, both within and outside the UK, attended the workshops (27 attendees at workshop 1 and 23 attendees at workshop 2). They gave valuable feedback on our stakeholder map and completed the follow-up questionnaire, providing information on their individual and organizations’ interest, influence and expertise in the bioaerosol field and views on knowledge gaps, future priorities and what could be gained from stakeholder mapping and engagement. The follow-up questionnaire was answered by 25 individuals who were invited to the workshops and who already had an interest in bioaerosols. This is reflected in the interest and influence scores (Material SD), whereby the majority (88–95%) gave an interest score of 5 out of 10 or more (whether completing scores from an individual or organizational perspective). Generally, responses indicated that organizations had more influence compared to individuals, highlighting the value of greater collective power in providing greater resources, larger networks, the ability to leverage the combined efforts of multiple individuals and thus the value of successful stakeholder mapping and engagement.

#### Expertise

The majority of respondents who attended the workshops (72%) reported their current interests to be in bioaerosol measurement and characterization, followed by public outreach, policy/regulation (41%), exposure/occupational risk assessment (34%) and toxicology/health effects (28%), noting that respondents could report more than one interest. When asked about desired interests for the future, the distribution was reversed (i.e. most interest extended to toxicology/health effects), which demonstrated that the respondents recognized a wide range of disciplines within bioaerosols and were keen to cross interdisciplinary borders. When asked about the types of bioaerosols of interest, 56% of attendees reported bacteria and viruses, 56% fungi and 38% pollen. This shows that there was a fairly equal distribution of interest across bacteria, viruses, fungi and pollen (the four key micro-organisms found in bioaerosols), suggesting that there was minimum bias in this respect. Historically, there has been greater a focus on bacteria and fungi; however, the COVID-19 pandemic resulted in a significant increase in research on airborne viruses. This, along with the tragic death of Awaab Ishaak, demonstrates how significant or well-publicized events can change the nature of stakeholder relationships and interest. Recognizing that bioaerosols are part of a wider airborne environment, additional stakeholders interested in other aeroallergens, such as house dust mite, animal dander or plant pathogen spores, would provide further valuable expertise.

Table 2 presents an overview of expertise represented by the individuals who attended the workshops and completed the follow-up questionnaire. The most dominant area of expertise was aerosol science, primarily represented by individuals with instrument, measurement and fieldwork experience, followed by microbiology. The least represented area of expertise was healthcare. The most available areas of expertise amongst the organizations represented at the workshop were data science, atmospheric science and dispersion modelling. The area of expertise of least availability was instrument manufacture. The most desired areas of expertise were healthcare, regulation and policy, which, along with instrument manufacture, had greater demand than availability. The other areas of expertise reportedly had availability to meet demand, provided that the appropriate connections are made. Again, this demonstrates that the attendees were aware of key skill gaps and keen to bridge them by bringing other expertise on board. Such information will help guide future engagement priorities.

**Table 2.** Expertise of the individuals who attended the workshops ('individual expertise’), the expertise that they had available in their organizations (‘available expertise’) and the expertise that they or their organization lacked that could benefit their work (‘desired expertise’)

Field of expertise	Individual expertise	Available expertise	Desired expertise
Atmospheric science	**7**	**17**	**3**
Aerosol science	**10**	**15**	**3**
Chemistry	**6**	**8**	**2**
Instrument/measurement/fieldwork experience	**10**	**15**	**2**
Toxicology	**2**	**11**	**8**
Microbiology	**8**	**12**	**6**
Instrument manufacture	**3**	**5**	**7**
Clinical care (e.g. consultant)	**1**	**7**	**3**
Engagement and outreach	**6**	**12**	**5**
Regulation/policy	**2**	**9**	**10**
Epidemiology	**5**	**11**	**6**
Healthcare	**0**	**7**	**10**
Machine learning	**4**	**11**	**6**
Data science	**7**	**18**	**7**
Dispersion/exposure modelling	**4**	**16**	**8**

#### Primary knowledge gaps and future focus areas

The primary knowledge gaps identified were as follows:

- *Need for improved monitoring*
  Discussions were primarily focussed on improving the monitoring of bioaerosols in ambient air, particularly the need for automated real-time monitoring networks across the UK. These were highlighted as vital for improving the spatial and temporal resolution of monitoring data to characterize bioaerosols in different environments, as well as providing better alerts for allergy sufferers and their healthcare providers (e.g. to improve the forecasting and communication of high bioaerosol levels). Other comments included the need to better understand the differences in current common sampling methods, speciation (see below) and what impacts the potency of pollen grains and improve ambient monitoring of viruses. Health monitoring was not specifically mentioned.
- *Improved data collection and quality*
  This involved consideration of the need for better designed exposure assessment studies (including improved dispersion modelling). Whilst the focus was mainly related to outdoor bioaerosols, indoor and occupational exposure were also mentioned. The difficulties with the measurement and detection of bioaerosols were acknowledged as a serious technical challenge making exposure assessment difficult.
- *Establishing clear links between exposure and health*
  The importance of better understanding the underlying biological mechanisms linking exposure and different health outcomes via *in vitro* and ex vivo studies, including assessment of the dose required to trigger a health response, was discussed. Characterization of bioaerosol composition was highlighted as a key part of assessing relationships between specific exposures and health outcomes (see below). The need for such data to inform future regulation and policy was acknowledged (see below).
- *Regulatory implications, policy implementation and establishment of guidance/threshold values*
  The need for evidence-based limits/guidelines/threshold values relating to health impacts was mentioned by many. It was thought that a specific and quantified understanding of dose-response relationships for susceptible individuals and populations was essential to promote the development and implementation of evidence-based policies and regulations. The need to engage with health professionals to examine dose-response relationships is an ongoing gap that was also discussed in [33]. Regulation of anthropogenic sources was also mentioned, with respect to how a more comprehensive assessment of the spatial and temporal variation of ambient bioaerosols is required to determine the contribution of anthropogenic sources to overall bioaerosol composition and levels. Specifically, one respondent mentioned the need for threshold values for airborne allergenic pollen and fungi, which can initiate allergic rhinitis and asthma, respectively. The ability to set these was considered crucial for public and occupational health, particularly within indoor environments.
- *Characterization of composition*
  Discussions focused on the importance of characterizing the specific taxa and their components within bioaerosols from different outdoor and indoor environments to help identify those of concern for negative health impacts, including pollen and fungal allergy. Differentiation between grass pollen species was specifically mentioned by two respondents. The need to better understand sub-pollen particles and other bioaerosol fragments was also highlighted, as these are not well understood but potentially have impacts on human health.

Other knowledge gaps identified (Material SE) included the following: (i) better understanding of the role of climate change and climatic variables on bioaerosol concentrations and seasonality, including what causes seasonal variability and how to better predict it; (ii) mitigation, particularly solutions to mitigate bioaerosols from anthropogenic sources and indoors, including strategies for ventilation and cleaning of the air and surfaces; (iii) better risk communication, including improved public awareness and pollen information for allergy sufferers and healthcare professionals (e.g. allergologists).

The areas identified as key to focus on include the following:

- *Standardized methods (sampling and analysis of bioaerosols) and research frameworks* – essential for integrating and comparing results across different studies and establishing international monitoring networks. End-to-end workflows incorporating the latest technologies and flow diagrams to select the most appropriate methods to sample and characterize bioaerosols for a particular application or research question were specifically mentioned.
- *Data integration and interdisciplinary collaboration* – including better linkage to European research communities.
- *Toxicological and epidemiological studies* – to help establish robust links between specific bioaerosol exposures and health outcomes, including benefits as well as harms. Focus on studies that consider fungal and pollen species that have not been considered previously was also suggested.
- *Better public messaging and dissemination tools* – to enable susceptible individuals and populations and healthcare professionals to better manage symptoms and prepare for greater demands on healthcare services when bioaerosol levels are high.
- *Focused future engagement and research* – to influence those with little interest but high power and better direct research to stakeholders’ needs.

#### Use of stakeholder mapping

Workshop attendees communicated that they would like this mapping and engagement to help keep future work focused to maximize impact and answer priority questions from regulators and policymakers to ensure evidence-based decisions and develop improved guidance. Cross-sector multi-disciplinary collaborations were considered key, coupled with improved outreach and influence, particularly to the public and funding organizations, by clarifying why these issues need to be solved and demonstrating what is both possible and feasible. It was deemed valuable to define the links and convene stakeholders from various perspectives so that their priorities can be aligned as closely as possible to maximize outputs via combined efforts. It was thought that such collaborative partnerships would have positive impacts by engaging the public with clear and pragmatic messages and persuading funders that it is an area worth investing in. The stakeholder map could then be used for identifying target audiences or new collaborative partnerships and developing appropriate communication and research dissemination approaches or funding applications. For example, the map would be useful at the beginning of the research cycle where an advisory board is being established, and specific sector group representatives are required. It could help develop communication strategies for research outcomes or opportunities to identify or target the next users of the information. Such maps could also be beneficial for identifying resource flows, detailing from where stakeholders can access certain data, expertise or funding.

Whilst this stakeholder mapping and its associated discussions were within the context of bioaerosols, the process and outcomes are also useful and relevant to other fields. Indeed, others have used similar questionnaire- and workshop-based approaches to map stakeholders within specific healthcare [26], citizen science [38] and health inequality [39] projects. We have taken this a step further by demonstrating the value of doing this for a whole field, not just a single project.

### Stakeholder mapping

**Fig. 4.**
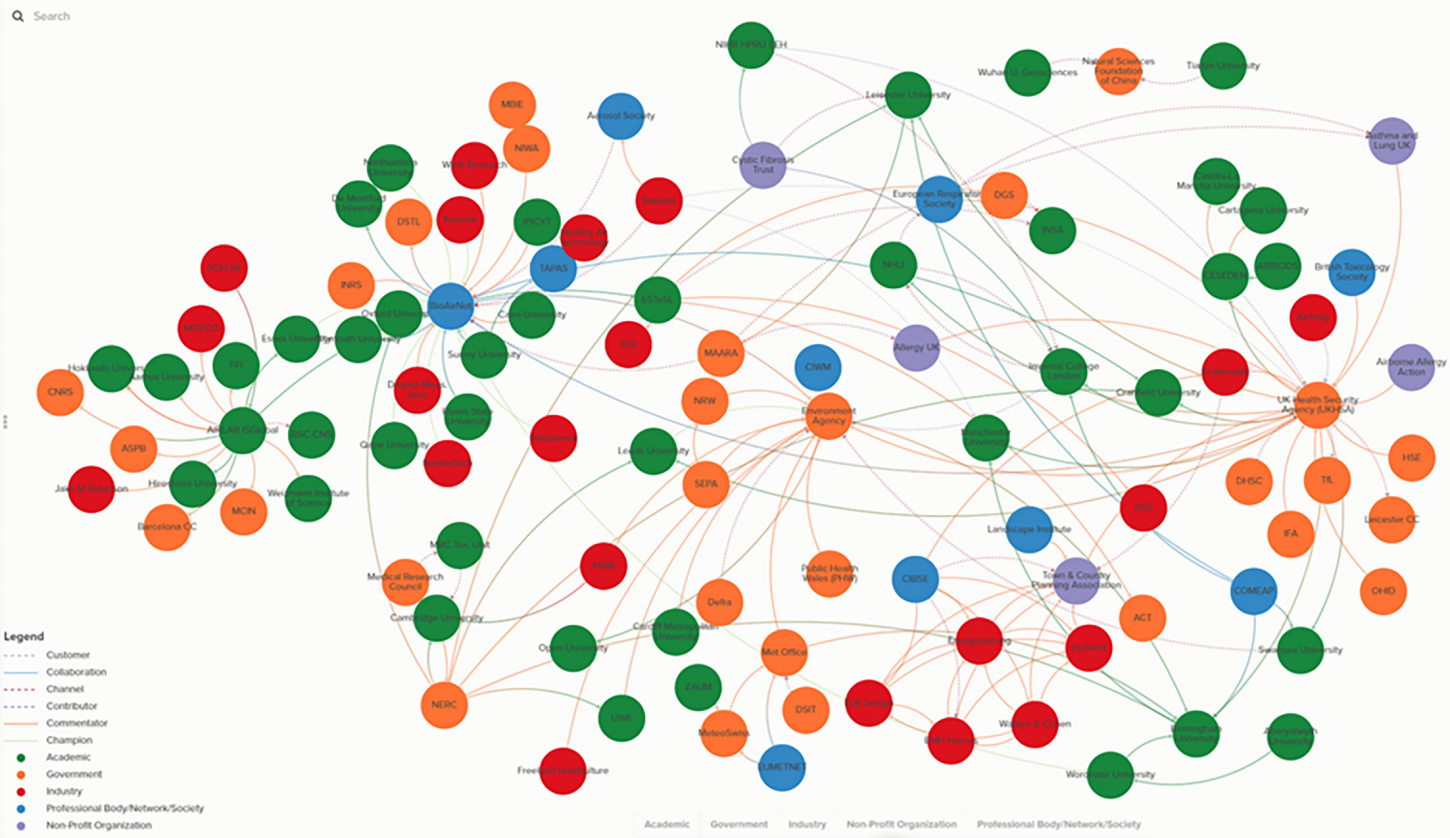
Stakeholder relationships, mapped using Kumu, utilizing the results from the questionnaires and workshops. Stakeholders were colour-coded by sector: academic (green), industry (red), government (orange), professional body/network/society (blue) or NPO (purple). The relationships were categorized using the 9Cs (defined in Table 1) into customer (lilac dashed line), collaborator (blue solid line), channel (dark-red dashed line), contributor (purple dashed line), commentator (orange solid line) or champion (green solid line). The map above is intended for illustrative purposes only. The full interactive version can be found here.

**Fig. 5.**
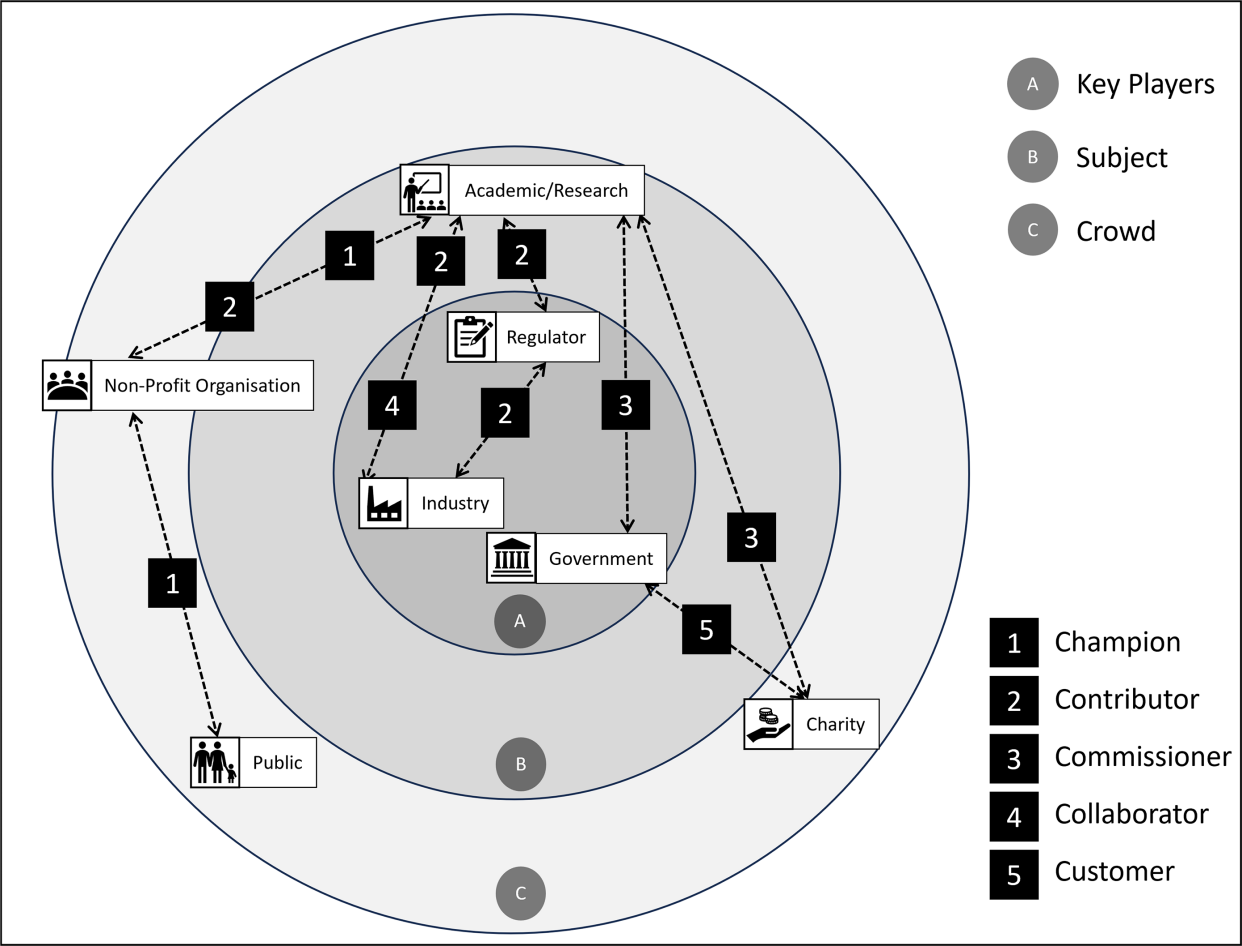
Stakeholder relationships shown graphically using an onion diagram [40], with stakeholder engagement types categorized using the 9Cs (defined in Table 1).

Mapping stakeholder relationships into an interactive network using Kumu (Fig. 4) or onto a layered onion diagram (Fig. 5) provided a way of visualizing and exploring links between stakeholders. The interactive Kumu-generated network enabled stakeholder relationships to be viewed by sector and the type of relationship, highlighting which sectors were well connected, where new relationships were required and which common contacts could facilitate links. It has been suggested that mapping stakeholders’ relationships provides more value and understanding than the final maps themselves [40]. The use of interactive and editable tools such as Kumu ensures that the maps can evolve with the field and helps to prevent them from becoming obsolete, if the owners regularly review and update.

The onion diagram illustrated the importance (and thus potential influence and interest) of stakeholder sectors, distinguishing the key players with a high level of interest and influence from subjects with high interest but minimal power and the crowd that has less influence and interest. Prioritizing engagement with key players has been suggested to help drive change [35]. Subjects can be excellent allies, providing support for gaining acceptance of, and interest in, the field/project. They should be regularly engaged, for example, through review and input sessions to keep them interested and involved [36]. Since the crowd has less influence and interest, it is argued that occasionally informing them of significant events and outcomes is all that is required [37]. However, to co-create fit-for-purpose policy change, researchers may foster greater collaboration with the public (crowd), as well as government/regulators (key players). Both visualizations improved understanding of how relationships between stakeholders may impact on stakeholder interests, perspectives and priorities and could be used to inform future communication and engagement strategies, as well as social network analysis [41] and/or strategic foresight analysis [42].

It is important to note that these maps are based on the results of the questionnaires and workshops. They are not a final complete picture of reality. These maps should be seen as live documents that evolve as the stakeholders expand and relationships between them grow or change.

### Strengths, limitations and future development

Whilst we endeavoured to include a wide range of stakeholders in the initial survey, we recognize that our approaches were biassed towards those already in the bioaerosol field and that it would be very challenging to include all stakeholders. Specifically, stakeholders representing the public and healthcare practitioners were not captured because they were not reached by the survey distribution method or did not have enough interest to respond. Workshop attendees recognized their biases and highlighted greater engagement with the agriculture, water and recycling industries; those interested in protecting occupants from low-quality housing and vulnerable people; clinicians and healthcare professionals; the general public; and more global stakeholders (specifically from the ‘Global South’), which was needed. We also did not specifically ask workshop respondents about some specific bioaerosol constituents, such as plant pathogen spores and house dust mites, therefore missing additional key groups of stakeholders.

In the future, we could make use of broader official stakeholder engagement channels to ‘recruit’ key industries and public interest groups and present this work at relevant multidisciplinary or cross-government meetings to raise wider awareness and interest. Engagement strategies targeted at missing sectors, groups and regions would also be an option. We have already started to engage with the public through an additional BioAirNet [31] workshop exploring public awareness and opinions on the health impacts of bioaerosols, and how to better communicate fundamental concepts. Suggestions arising from that workshop included engaging journalists and media organizations, educating health professionals, incorporating activities into the school curriculum, more citizen science and engagement projects, creating tangible evidence and refining key messages. As part of BioAirNet, we have already developed various resources for schoolchildren, including online materials for teachers covering multiple key stages [43] and activities for British Science Week 2024 [44]. It is important to remember that ‘the public’ should not be treated as a single stakeholder, as they are a diverse group with different perspectives and needs. Further analysis of this particular stakeholder group should be undertaken to define this more explicitly, supported by further outreach activities using communication strategies suitable for more lay audiences. The stakeholder map developed here could be used to facilitate knowledge exchange, mobilization of research findings, collaboration, education or raise awareness and therefore may be valuable to target different groups.

We were unable to fully capture existing connections, collaborations, institutional links and flows of interest between stakeholders. Workshop attendees agreed that such networks were useful for identifying new contacts and potential collaborators, as well as understanding how different groups fit together. The stakeholder diagrams and maps that we created were also unable to fully capture the presence and direction of resource flows, including data, skill sets and funding, as well as power dynamics amongst stakeholders. Further thought will have to be given in the future on how to capture and incorporate these aspects into a deliverable map.

Whilst we have made a significant step forward here, presenting the work at multiple conferences has highlighted that we could better explore stakeholder relationships in the future with social network analysis [41]. Identifying and engaging with other networks, such as the UKHSA stakeholder engagement channels, to participate further in this work would be a key future development. In addition, developing plans to present this work in different conferences and cross-government meetings will expand the input into the stakeholder maps.

Another difficulty encountered with this study was the difference of subjectivity across stakeholders. For example, the engagement types, interest and influence scores were dependent on the perspective that stakeholders were answering from, e.g. as an organization, department, team or individual, since this was not clearly defined in the initial survey. The answers that they gave may also have varied subjectively across different people within the same organization depending on their individual understanding and views. The target of ‘bioaerosols’ was also very general and therefore difficult to summarize engagement types, interest and influence scores. It was difficult to standardize this data, and future work may benefit by better defining the questions and specifying from what perspective the stakeholders should respond (their own or their organization’s perspective). There may also be additional bias in the perceived and potentially varied understanding of influence and interest of the respondents. We would advocate mapping the stakeholders across a whole field and then focussing in on specific topics, issues and stakeholders with additional engagement activities.

The potential impact of future changes (such as climate change, government policies or regulation) on stakeholder relationships and their influence and interest could also be explored. A useful approach here would be the use of strategic foresight to develop future scenarios. The Axes of Uncertainty/Scenario Axes technique, as used by Shetty [45], allowed for the examination of various future scenarios. This takes into account regulatory dynamics, economic situations and policy changes and could therefore be a useful approach to consider how the stakeholders and their characteristics might evolve. This will improve the usefulness of the analyses presented here, making it more applicable to a wider range of scenarios and stakeholders.

## Conclusions

The benefits and uses of stakeholder mapping are being increasingly realized across scientific research. Here, we have presented, to our knowledge, the first such analysis for the bioaerosol research field. Bioaerosol research, due to its complexity, relies on many disciplines, which have wide-ranging impacts, and this approach has allowed for the diverse range of stakeholders to be identified and mapped for the first time.

This analysis has been undertaken by UK-based bioaerosol research scientists within academia and government. This should therefore provide an example and encouragement to others to adopt this approach to further their understanding of their own research fields. We hope that by sharing our experience, others will see the value in this, and we hope to engage further with experts in stakeholder analysis and knowledge mobilization to develop this further.

The assessment of interest and influence has identified where we need to focus future engagement strategies to help raise awareness and facilitate knowledge mobilizzation.

In summary, taking the time to comprehensively identify, characterize and engage with our stakeholders has enabled us to identify missing stakeholders and expertise from our networks, particularly the public and healthcare professionals.

## Supplementary material

10.1099/mic.0.001574 Uncited Supplementary Material 1.

## Abbreviations

NPOs: non-profit organizations
