# Supplementary material for: Use of bioaerosol stakeholder mapping and engagement for the development of future strategic collaborations: a UK perspective
Source: Microbiology (Reading). 2025 Jul 16;171(7):001574. doi: 10.1099/mic.0.001574 (PMC12282280; doi:10.1099/mic.0.001574)
Supplement: Uncited Supplementary Material 1. [file mic-171-01574-s001.pdf]

Supplementary Material for:

Use of bioaerosol stakeholder mapping and engagement for the development of future strategy: A UK perspective

Sophie A. Mills[1,2], Sana G. Shetty[1], Emma-Jane Goode[3], Gill Drew[1], Kerry A Broom[3,4,5,6], Emma Marczylo[3,5,7], Pippa Douglas[3,7,8]

1. Cranfield University, College Road, Wharley End, Bedford, MK43 0AL, UK
2. School of Geography, Earth and Environmental Sciences, University of Birmingham, Birmingham, B15 2TT, UK
3. Radiation, Chemical, Climate and Environmental Hazards, UK Health Security Agency, Chilton, Didcot, Oxfordshire, OX11 0RQ, UK
4. NIHR Health Protection Research Unit in Chemical Radiation Threats and Hazards
5. NIHR Health Protection Research Unit in Environmental Exposures and Health
6. NIHR Health Protection Research Unit in Environmental Change and Health
7. Centre for Environmental Health and Sustainability, University of Leicester, Leicester, UK
8. Chief Scientist's Group, Environment Agency, Red Kite House, Wallingford, UK

Contents

Supplementary Material A: Original questionnaire questions (Microsoft Forms) 2

Supplementary Material B: Workshop invitation and agendas 4

Supplementary Material C: Follow-up questionnaire questions (Qualtrics) 8

Supplementary Material D: Interest and influence results from the follow-up questionnaire 11

Supplementary Material E: Other results from the follow-up questionnaires and workshops 12

Supplementary Material A: Original questionnaire questions (Microsoft Forms)

1. Please provide your email address and affiliations (free text)
2. What is the name of the Stakeholder (organisation or individual)? (free text)
3. Is the stakeholder an existing stakeholder or a future stakeholder? Options:
    - Existing
    - Future
4. What sector is the stakeholder? Options (>1 could be selected):
    - Local Government
    - National Government
    - Regulator
    - Academic/Research
    - Charity
    - Industry
    - Professional Body
    - Press/Media
    - Other (please state)
5. What is the reason for engagement? (free text)
6. What is the frequency of engagement? Options:
    - Weekly
    - Monthly
    - Annually
    - Ad hoc
7. What is/are the current engagement type(s)? Options (>1 could be selected, and definitions (see Table 1) were provided):
    - Commissioner
    - Customer
    - Collaborator
    - Contributor
    - Channel
    - Commentator

- Consumer
- Champion
- Competitor

8. Is the future engagement type(s) the same as the current engagement type? Options:

- Yes
- No (the engagement types in question 7 reappeared)

9. What is the stakeholder's level of influence? Influence indicates relative power over and within a project, or ability resist recommendation or change:

- 1 (Low influence)
- 2
- 3
- 4 (High influence)

10. What is the stakeholder's level of interest? Interest indicates stakeholders' likely concerns, but could also be benefits for the stakeholder:

- 1 (Low interest)
- 2
- 3
- 4 (High interest)

11. Do you have any other information or comments to add for this stakeholder? (free text)

12. Do you have another stakeholder to add? Options:

- Yes (If yes the form repeated from question 2 for the additional stakeholder)
- No

13. Do you have any other comments? (free text)

Supplementary Material B: Workshop invitation and agendas

Bioaerosols Stakeholders Workshops

Workshop 1: Monday 9th Oct 2023 (10:00-12:00 GMT) online
Workshop 2: Monday 16th Oct 2023 (10:00-12:00 GMT) online

Bioaerosols encompasses the huge variety of particles in the atmosphere of biological origin. They are ubiquitous, being generated from both natural sources and anthropogenic activities, and can have complex interactions with humans, the environment, and the climate. While exposure to a diverse range of microbes is key to normal development of the immune system, exposure to higher levels of specific bioaerosols can negatively impact human health. The percentage of people who suffer from allergies including hay fever and asthma is increasing, there have been fatalities from unhealthy conditions of indoor mould, and the whole world has experienced the devastating effect that an airborne pathogen such as COVID-19 can have on our health, economy, and wellbeing. Yet there is still much progress to be made, for example, characterising these particles in ambient air, fully understanding their contributions to human and environmental health and the climate, and educating the public.

With increasing interest in bioaerosols over recent years, there is great benefit in establishing key stakeholders and their influence on funding, research, policy, and action relevant to this area. Mapping stakeholders and their associated relationships

would facilitate improved communication, multidisciplinary collaboration, and progress in addressing the issues at hand.

These workshops will:

1. Share the results of a comprehensive stakeholder mapping exercise based on data collected from our preliminary survey sent out to various stakeholders.
2. Collect further information and feedback to refine and improve the stakeholder map and discuss how it may be useful to the bioaerosols community going forwards.

Stakeholders will be encouraged to discuss and share perspectives to develop a roadmap for future progress. Additionally, this should present an opportunity for stakeholders to make useful connections and build potential collaborations.

Objectives:

- Review draft stakeholders map:
    - Distinguishing between current and future stakeholders.
    - Highlighting any gaps in terms of stakeholders, knowledge and/or expertise.
- Collect further information via follow-up survey focusing on stakeholders' various expertise and interests to facilitate productive connections and potential collaborations between stakeholders who share interests and/or have complementing expertise.
- Discuss and share perspectives on how this stakeholder map can be useful to the bioaerosol community and develop a roadmap for future progress.

Programme for Monday 9th Oct 2023

10:00-10:15	Welcome.<br>Introductions and purpose of workshop.
10:15-10:30<br>10:30-10:35	Review of results from initial survey & analysis.<br>Questions.
10:35-10:45	Break
10:45-10:55	Introduce follow-up survey and explanation of how data will be used.
10:55-11:00	Introduction to breakout room discussions & Jamboards.
11:00-11:30	Breakout rooms:<br>Discussion of survey questions and what else might be useful information to gather or topics for discussion.

11:30-11:45	Networking session (during discussion between facilitators).
11:45-12:00	Summary of points discussed during breakout sessions from facilitators.<br>Closure & notes for next workshop.

After the first workshop, participants will be encouraged to complete the survey if they have not already. The additional information collected from this survey will be presented at the second workshop and points raised from discussions (recorded on online whiteboards) during the first workshop will be used to focus discussions at the second workshop.

Programme for Monday 16th Oct 2023 (TBC)

10:00-10:10	Re-introduction and quick reminder of last workshop
10:10-10:30	Summary of results from follow-up survey.
10:30-10:45	Reflection and further thoughts from stakeholders after last workshop and survey.
10:45-11:00	Breakout rooms:<br>Discussion of topic/question TBC
11:00-11:10	Break
11:10-11:25	Breakout rooms:<br>Discussion of topic/question TBC
11:25-11:50	Summary of discussions and important points.<br>Discussion of roadmap and recommendations for future progress.
11:50-12:00	Thank you and final points.

This stakeholder mapping work and workshops are supported by BioAirNet (https://bioairnet.co.uk/) – the Indoor/Outdoor Bioaerosols Interface and Relationships Network, funded through the UKRI Strategic Priorities Fund (SPF) Clean Air Programme – as a joint effort with collaborators at Cranfield University, the UKHSA and the Environment Agency. The facilitators who will lead the workshops and their affiliations are listed below:

Facilitators:

**Name**	**Affiliation**
Sophie Mills	Cranfield University (BioAirNet)/<br>University of Birmingham
Emma Marczylo	UKHSA
Pippa Douglas	Environment Agency
Kerry Broom	UKHSA
Gill Drew	Cranfield University (BioAirNet)
Sana Shetty	Cranfield University

Supplementary Material C: Follow-up questionnaire questions (Qualtrics)

1. Please enter your name. (free text)
2. Please enter the name of the organisation you are representing. (free text)
3. Please enter your email. (free text)
4. Which of the following aspects of bioaerosols does your work primarily involve? Options (>1 could be selected):
   - Bioaerosol measurement & characterisation
   - Exposure/occupational risk assessment
   - Toxicology/Health effects
   - Public outreach/policy/regulation
   - Other (State)
5. Which are you not primarily involved in but interested in? Options (>1 could be selected):
   - Bioaerosol measurement & characterisation
   - Exposure/occupational risk assessment
   - Toxicology/Health effects
   - Public outreach/policy/regulation
   - Other (State)
6. Which types of bioaerosols do your interests cover? Options (>1 could be selected):
   - Pollen
   - Fungal
   - Bacteria
   - Viral
   - Other
7. How would you describe your areas of expertise? Options (>1 could be selected):
   - Atmospheric scientist
   - Epidemiologist
   - Toxicologist
   - Health care practitioner

- Dispersion/exposure
- Modeller
- Instrument manufacturer
- Aerosol scientist
- Data scientist
- Machine learning practitioner
- Microbiologist
- Chemist
- Instrument/measurement/fieldwork expertise
- Engagement & outreach
- Consultant
- Regulator/policymaker
- Other (State)

8. How would you describe the areas of expertise available to you within your team and collaborators? Options (>1 could be selected):

- Atmospheric scientist
- Epidemiologist
- Toxicologist
- Health care practitioner
- Dispersion/exposure
- Modeller
- Instrument manufacturer
- Aerosol scientist
- Data scientist
- Machine learning practitioner
- Microbiologist
- Chemist
- Instrument/measurement/fieldwork expertise
- Engagement & outreach
- Consultant
- Regulator/policymaker
- Other (State)

9. What areas of expertise or skills don't you have access to that could benefit your work?

- Atmospheric scientist
- Epidemiologist
- Toxicologist
- Health care practitioner
- Dispersion/exposure
- Modeller
- Instrument manufacturer
- Aerosol scientist
- Data scientist
- Machine learning practitioner
- Microbiologist
- Chemist
- Instrument/measurement/fieldwork expertise
- Engagement & outreach
- Consultant
- Regulator/policymaker
- Other (State)

10. How would you score the following (Low 0- High 10)?

- Your personal interest towards bioaerosols?
- The general interest of your organisation towards bioaerosols?
- The level of influence your organisation has in this area?
- The level of influence you have as an individual in this area?

11. Do you offer funding? Options:

- No
- Sometimes - e.g. small pump priming/pilot grants
- Yes - large project grants.

12. What are the primary knowledge gaps concerning bioaerosols and important aspects to focus on to facilitate future progress? (free text)

13. What questions do you think are important to discuss among stakeholders? (free text)

14. What would you like to gain from stakeholder engagement? (free text)

Supplementary Material D: Interest and influence results from the follow-up questionnaire

The influence and interest scores are presented graphically in Figure D1(A) when completed from an organisational perspective, and Figure D1(B) when completed at an individual perspective. Scores were provided on a scale of 1-10 (10=high).

A

B

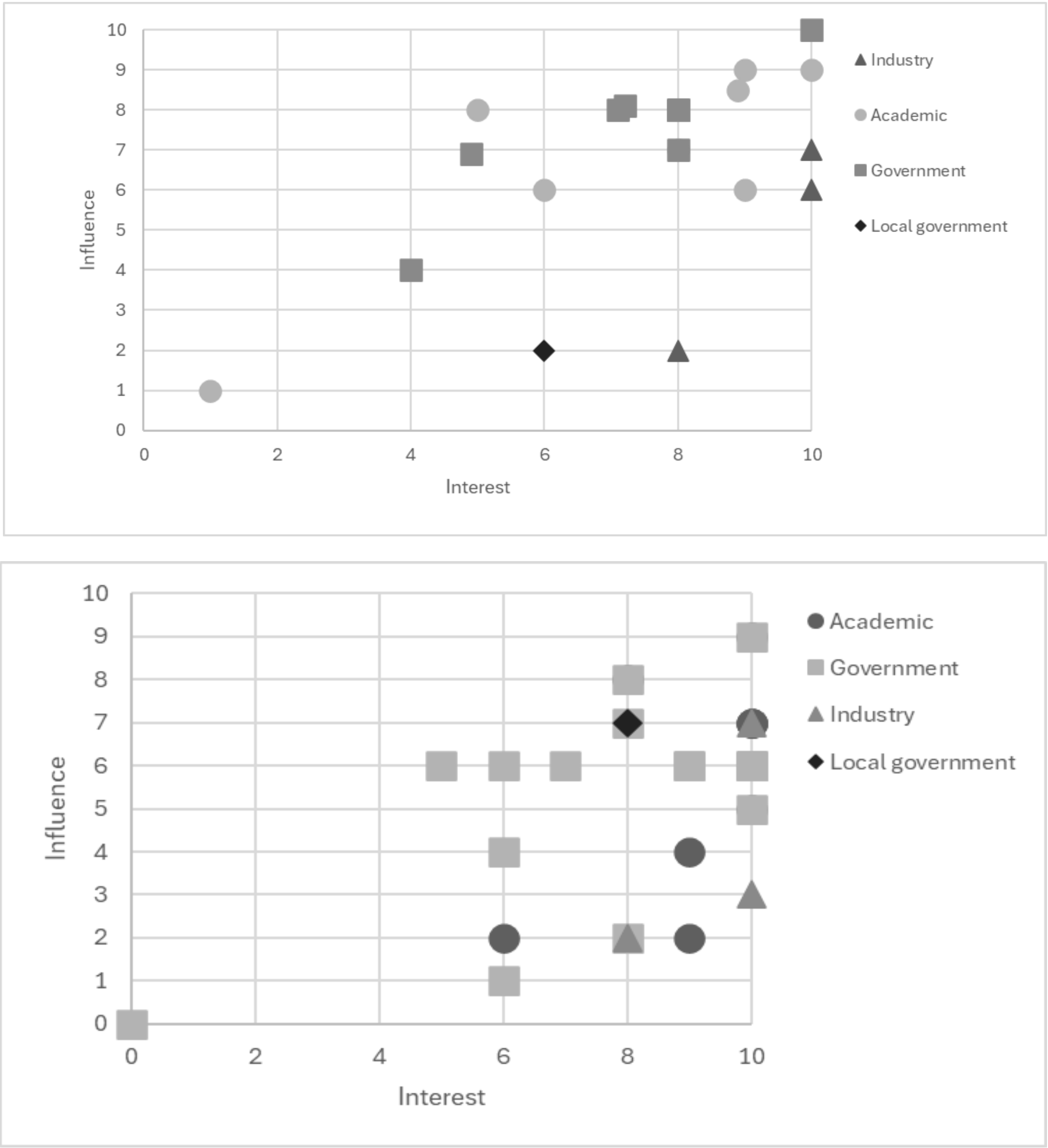

Figure D1. The influence and interest scores when completed from an organisational perspective (A), and an individual perspective(B). Scores were provided on a scale of 1-10 (10=high).

Supplementary Material E: Other results from the follow-up questionnaires and workshops

Is funding offered?

In the follow-up questionnaire, a question was asked whether the organisation that the individuals represented offered any funding (see question 11, Supplementary material C). Of the 34 people that responded, 0% (n=0) said ‘Yes’, 26% (n=9) said ‘Sometimes’, 62% (n=21) said ‘No’ and the remaining 12% (n=4) did not respond to the question.

How do we facilitate skills sharing and collaboration and create a roadmap for the future?

Raising awareness was a strong theme and how there needs to be more openness and transparency. This applies to the experts working on bioaerosols and data availability. Co-creation and collaborative working were encouraged, and the idea of a ‘what can I offer’ summary from participants in the workshop could be used to raise awareness. Regulators industry and policy links were also considered important.

The use of existing networks and contacts should be considered to increase collaboration and use existing data and networks to demonstrate impact, as well as making good use of conferences and mapping stakeholder outputs.

The idea of networks/focus groups were strongly suggested especially in specific detailed subject areas e.g, monitoring or standardisation of measurements, but there was acknowledgment that some data and information could be sensitive.

There was also a recognition that more research is required especially that bioaerosols research is complex, and the health effect mechanism are not known. There is also complexity of exposure and response and how we get the right people to get involved in this work.

How do we engage with public, health practitioners and other stakeholders to increase awareness and interest?

For engagement with the public, the use of citizen science through e.g., monitoring apps or low-cost sensors was discussed, and outreach events through festivals e.g., British Science Week, with families and schools were thought to be beneficial.
It was thought advantageous to work alongside journalists or the Science media centre to help with resources.

There was also a suggestion to undertake a survey to capture general attitudes and awareness of public, as the survey itself can be used as a communication tool to raise their awareness and interest.

Local authorities/health providers engagement focussed on the education of colleagues on the importance of bioaerosols and allergies. This could be done through focus groups or workshops, but also embedded in training on environment and health. Using existing health charities and allergy clinics could also prove fruitful.

For engagement with other stakeholders, the use of existing contacts to reach wider contacts, especially those in HPRUs and use of the outreach networks. Environmental consultancies should also be engaged with, as well as those linked to indoor air quality, civil engineering and building organisations. Cross cutting topics, that may impact outdoor and indoor air pollution such as climate change, or damp/mould, or cost of living could be used as an anchor to begin discussions about aerosols with different audiences. Many charities and stakeholders from other sectors could be engaged. resources/activities, perhaps with BioAirNet as a good starting point. There are some internal data sharing systems that could be rolled out, such as through UKHSA and their Environmental Public Health Surveillance System.

Consideration was also made to stakeholder engagement outside of the UK, and how different European countries are lobbying parliaments and how we can learn from best practice. It was noted that evidence needs to be tangible so that can be embedded in practise. Position papers or regular newsletters were thought to be good ways of engaging with stakeholders, and highlighting why they should be interested.

Key messages should be agreed upon to avoid misinformation or conflicting information.
